# Atom Efficient Preparation of Zinc Selenates for the Synthesis of Selenol Esters under “*On Water*” Conditions

**DOI:** 10.3390/molecules22060953

**Published:** 2017-06-08

**Authors:** Luca Sancineto, Jaqueline Pinto Vargas, Bonifacio Monti, Massimiliano Arca, Vito Lippolis, Gelson Perin, Eder Joao Lenardao, Claudio Santi

**Affiliations:** 1 Catalysis and Organic Green Chemistry Group, Department of Pharmaceutical Sciences, University of Perugia, Via del Liceo 1, 06100 Perugia, Italy; sancineto.luca@gmail.com (L.S.); bonifaciomonti@gmail.com (B.M.); 2 Universidade Federal do Pampa—Unipampa, Av. Pedro Anunciação, 111, Caçapava do Sul-RS 96570-000, Brazil; jaquelinevargas@unipampa.edu.br; 3 Dipartimento di Scienze Chimiche e Geologiche, Università degli Studi di Cagliari, S.S. 554 bivio per Sestu, 09042 Monserrato, Cagliari, Italy; marca@unica.it (M.A.); lippolis@unica.it (V.L.); 4 Laboratório de Síntese Orgânica Limpa-LASOL-CCQFA, Universidade Federal de Pelotas-UFPel, P.O. Box 354, Pelotas-RS 96010-900, Brazil; gelson_perin@ufpel.edu.br

**Keywords:** selenium, selenol esters, zinc, TMEDA, water

## Abstract

We describe here an atom efficient procedure to prepare selenol esters in good to excellent yields by reacting [(PhSe)_2_Zn] or [(PhSe)_2_Zn]TMEDA with acyl chlorides under “on water” conditions. The method is applicable to a series of aromatic and aliphatic acyl chlorides and tolerates the presence of other functionalities in the starting material.

## 1. Introduction

**Figure 1:**
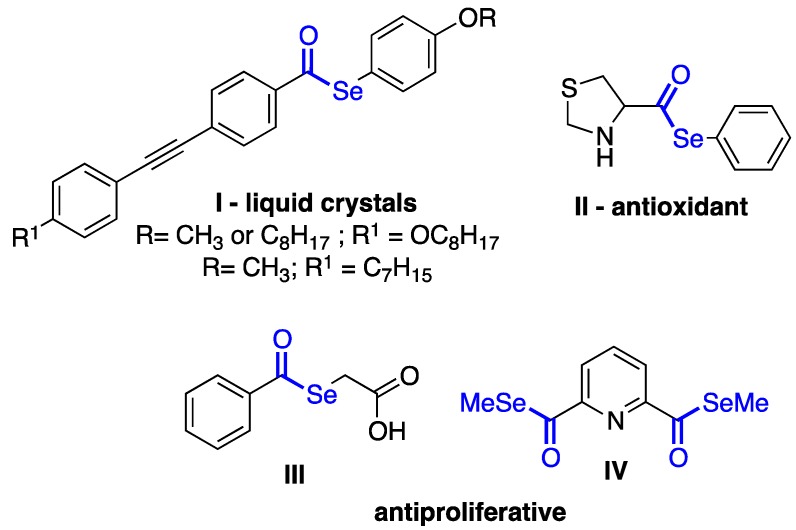
Examples of molecules bearing the selenol ester moiety.

Selenol esters are versatile tools in organic synthesis, once they can be easily converted to a sort of more complex molecules, acting as acyl-transfers, and in other important functional groups modifications [1]. Aromatic selenol esters, such as **I**, are stable liquid crystals with a large nematic mesophase range [2,3,4]. In contrast to their sulfur analogues, selenol esters only recently have attracted the attention to their pharmacological potential; the thiazolidine-4-carboselenoate **II** is a potent antioxidant [5], while the polyfunctionalized selenol esters **III** and **IV** presented high cytotoxic and antiproliferative activities against MCF-7 human cancer cells [6,7] (Figure 1).

The synthetic usefulness of selenol esters goes far beyond the transfer of acyl group [8], or its use as a protecting group for selenium compounds [9]. Selenol esters were explored in the total synthesis of several complex molecules [10,11,12,13,14], such as the marine alkaloid amphimedime [10], apparicine [11], (+)-geissoschizine [12], ciguatoxin CTX3C7d [13] and the peach moth (*Carposia niponensis*) pheromone [14]. More recently, selenol esters have gained even more importance, after the discovery that they can be used in native chemical ligation (NCL) reactions where the traditional thioester ligation chemistry is prohibitive [15,16].

As a consequence of the increasing demand for selenol esters, the number of methods to prepare this class of compounds has been increased along the years [1,17,18,19,20,21,22,23,24,25,26,27,28,29,30,31,32,33,34,35,36,37,38,39,40,41,42,43] Among the strategies to prepare selenol esters there are the reactions of nucleophilic selenium reagents with an acyl group source, such as *N*-acyl benzotriazoles [17,18] activated carboxylic acids (using DCC [3] or PBu_3_ [4,5,19,20]), enol esters [21], anhydrides [22,23,24], esters [25], carbon monoxide [26,27], aldehydes [28,29] or acyl chlorides [2,7,30,31,32,33,34,35,36,37,38,39,40,41,42,43]. Other approaches to selenol esters involve the alkylation of selenocarboxylate anions with alkyl halides [6,44,45,46] or the acidic hydration of selenoalkynes [47,48]. The acyl substitution of acyl chlorides is by far the most explored method to access selenol esters, mainly because of its versatility, since the diversity of acyl chlorides that can be prepared is practically endless. The pivotal step in this reaction is the generation of the nucleophilic selenium species and a range of reagents have been used for this purpose, including Se°/ArMgBr [2], (NH_2_)_2_C=Se/Et_3_N [30], Se°/NaBH_4_ [6], InI/(PhSe)_2_ [31,39,40], Se°/LiAlH_4_ [32,37], (RSe)_2_/Zn/AlCl_3_ [33], Ph(NH_2_)C=Se [34], (Bu_3_Sn)_2_/(PhSe)_2_/hv [35,36], Se°/R_2_C=CZrCp_2_Cl [38], (RSe)_2_/Zn°/[bmim]PF_6_ [41], (PhSe)_2_/Hg°/dioxane [42] and (PhSe)_2_/SnCl_2_/CuBr_2_/[bmim]BF_4_ [43]. Despite these reaction systems afforded a range of selenol esters, they suffer from one or more of the following drawbacks: use of VOCs as solvent, strong bases, strong and moisture sensible reducing agents, expensive reagents and low atom-economy.

Significant progress towards the greenness of the synthesis of selenol esters has been recently described [49,50]. Braga and co-workers described the solvent-free, microwave accelerated reaction of diorganyl diselenides with acyl chlorides; good yields of selenol esters were obtained after irradiation at 80 °C for only 2 min [49]. In the same year, some of us described the use of the bench stable nucleophilic species, PhSeZnX (X=Br, Cl), in the synthesis of a range of selenol esters in good yields at room temperature and using water as medium [50]. It was observed that the reaction was accelerated when it was performed under “on water” conditions and, in addition, the water was reused for subsequent cycles of reactions.

Nine years ago, we introduced a simple method to prepare in situ nucleophilic sulfur and selenium reagents by reducing dichalcogenides with elemental zinc in an acidic biphasic system [51]. This protocol has been more recently used by us and others to effect a series of selenylation reactions involving nucleophilic substitutions, ring opening and hydrochalcogenations [51,52,53] and it was adopted by Flemer in the synthesis of peptides [54,55].

The actual reactive specie in this protocol was supposed to be a zinc bis-selenate [(PhSe)_2_Zn] probably in equilibrium with the corresponding selenol. Considering that [(PhSe)_2_Zn] is characterized by a higher atom economy respect to the PhSeZn-halides in the insertion of PhSe groups, the possibility to obtain it in a bench stable form is desirable. It could improve the versatility of its synthetic application (avoiding the strong acidic conditions of the biphasic system), controlling or preventing undesired side reactions like those observed when it was prepared and used in situ in the presence of THF [56].

## 2. Results

To better understand and prevent the side reaction observed in the presence of THF [56], DFT calculations were performed comparing [(PhCh)_2_Zn] (Ch=S, Se) with the previously reported PhChZnX (Ch=S, Se; X=Cl, Br) [57].

**Figure 2:**
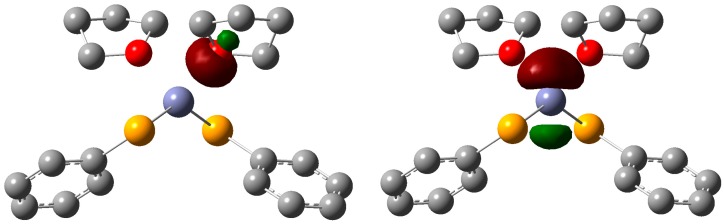
Drawings of the NBOs involved in the second order interaction between (PhSe)_2_Zn and the THF fragments in the compound (PhSe)_2_Zn·2THF at the DFT-optimized geometry (energy difference 0.7465 a.u.; interaction energy 25.77 kcal·mol^−1^). **Left**: filled NBO LP localized on the oxygen donor atom of one THF solvent fragment (NBO #127, 34.45% s and 65.54% p character). **Right**: virtual NBO LP* localized on the Zn center (NBO #124, 9.51% s and 90.35% p character). Cutoff value: 0.1 |e|. Hydrogen atoms have been omitted for clarity. (Grey = Carbon; Red = Oxygen; Yellow = Selenium; Violet = Zinc).

DFT-optimized geometries of the (PhCh)_2_Zn species (Ch=S, Se) show a nearly linear coordination at the Zn center. This geometry has been observed in the gas phase for ZnH_2_ and ZnCl_2_, [58] although several examples are reported of structurally characterized zinc(II) compounds featuring discrete linear E–Zn–E moieties with E=O [58,59,60], S [61,62], or Se [63]. On passing from Ch=S to Ch=Se, the natural charge polarization of the Ch–Zn bond decreases (1.233 and 1.093 e for Ch=S and Se, respectively), suggesting a higher reactivity of (PhS)_2_Zn in all conditions as compared to (PhSe)_2_Zn. The solvated species (PhCh)_2_Zn·2solv (solv=THF, H_2_O) were also successfully optimized. For both solvents, solvated species show a distortion of the Ch–Zn–Ch moiety towards the usually encountered tetrahedral coordination of the Zn center. A Second Order Perturbation Theory Analysis of Fock Matrix in NBO Basis allows evaluating the interaction energies between the Zn center and each solvent molecule by about 50, 58, 48, and 55 kcal·mol^−1^ for (PhS)_2_Zn·2THF, (PhS)_2_Zn·2H_2_O, (PhSe)_2_Zn·2THF, and (PhSe)_2_Zn·2H_2_O, respectively. Such interactions arise from the electron density donation from the oxygen LP of the solvent units to the antibonding empty Zn atomic orbitals (Figure 2) and, to a minor extent, to the Zn-Ch antibonding NBOs, and result in an overall electron transfer of 0.095, 0.105, 0.133, and 0.104 e for (PhS)_2_Zn·2THF, (PhS)_2_Zn·2H_2_O, (PhSe)_2_Zn·2THF, and (PhSe)_2_Zn·2H_2_O, respectively. Notably, the interactions between (PhCh)_2_Zn species and the solvent units are remarkably stronger than those calculated between PhChZnX (Ch=S, Se; X=Cl, Br and I) and the same solvents, which were calculated in about 18 kcal·mol^−1^ at the same level of theory.

All (PhCh)_2_Zn species and the corresponding solvated forms show their HOMOs largely localized on the negatively charged Ch atoms (*Q_S_* = −0.315, −0.377, and −0.367 |e| for (PhSe)_2_Zn, (PhCh)_2_Zn·2THF, and (PhCh)_2_Zn·2H_2_O; *Q*_Se_ = −0.236, −0.305, and −0.274 |e| for (PhSe)_2_Zn, (PhCh)_2_Zn·2THF, and (PhCh)_2_Zn·2H_2_O, respectively).

The LUMOs of (PhCh)_2_Zn species are antibonding MOs displaying a remarkable contribution from the zinc atomic orbitals. About solvated species, it is worth noting that both (PhS)_2_Zn·2THF and (PhSe)_2_Zn·2H_2_O feature empty molecular orbitals with large contributions from the virtual molecular orbitals of the solvent molecules, which are therefore sensibly stabilized. In particular, the LUMO+4 MO calculated for THF shows an antibonding character with respect to the C–O bonds Kohn-Sham (KS) eigenvalue ε = +0.1389 Hartree). This orbital contributes to the LUMO+14 and LUMO+15 of (PhS)_2_Zn·2THF and (PhSe)_2_Zn·2THF, respectively, which are stabilized in energy by 0.887 eV (ε = +0.1063 Hartree for both virtual MOs). The strength of the interaction between THF and (PhCh)_2_Zn accompanied by a low-lying MO with remarkable C–O antibonding character partly localized on the coordinated THF units may account for the ring opening reactions of coordinated solvents observed experimentally [56].

Therefore, we investigated the possibility to prepare an isolable zinc bis-selenate to be used in the synthesis of chalcogenol esters avoiding the presence of THF during the reaction with the acyl chlorides. Different conditions for the oxidative insertion of the zinc in the Se-Se bond starting from the commercially available diphenyl diselenide were explored (Table 1). The reduction of diselenides in organic solvents can be unequivocally evidenced by the discoloration of the originally yellow solution and it was observed that both the presence of catalytic amount of TFA (10 mol %) and the THF (or a 1:1 THF/water mixture) are mandatory for the reduction at reflux (Table 1, entries 4 and 5 respectively). Interestingly, the insertion did not occur in refluxing THF for 2 h (Table 1, entry 2) neither in water suspension at the same temperature (Table 1, entry 1), as well as in the presence of TFA (10 mol %) (Table 1, entry 3).

**Figure 3:**
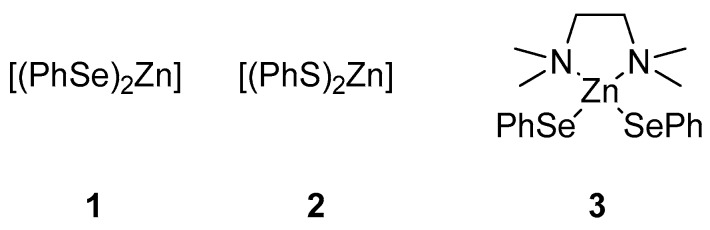
Reagents investigated in the present work.

Using the conditions depicted in Table 1, entry 4, after the discoloration, the THF was removed under-reduced pressure, giving a whitish amorphous and, unfortunately, relatively unstable solid, which was used without further purification. The supposed formation of a polymeric form of the reagent **1** was confirmed by the presence of a broad signal at −41 ppm in the ^77^Se NMR. Similarly, starting from diphenyl disulfide, the zinc bis-thiolate **2** was formed in situ (Figure 3). In this case, the starting material is colorless and the reduction time was arbitrarily chosen according to that of the reduction of diphenyl diselenide to afford **1**. A monomeric and more stable zinc selenate (**3**) can be prepared according to the literature using the bidentate TMEDA(*N,N,N^1^,N^1^*-tetramethyl-ethylendiamine) for the stabilization of the complex **3**, with TMEDA in the place of THF [64].

The reactivity of **1**,**3** and **2** with benzoyl chloride (**4a**) for the formation of the selenol ester **5a** or thiol ester **6a**, was evaluated in “on water” conditions (Table 2, entries 10–12) and these results were compared with some data recently reported using other zinc selenates, as well as different reaction conditions (Table 2, entries 1–3, 5–9). The bis-phenylselenate (**1**) showed an interesting reactivity affording, after 30 min, the formation of **5a** in 83% yield (Table 2, entry 10). This result confirms that zinc selenates, as previously reported, are efficient selenenylating reagents for the on-water acyl substitution. In addition, the use of water as medium for the reaction with the acyl chloride prevents the undesired ring opening of THF [56], affording diphenyl diselenide and benzoic acid as side organic products of residual decomposition, and TMEDA when **3** was used. Reasonably the zinc derivatives are removed during the workup due to their water solubility. We also observed that the reactivity of **1** in THF depends on the acid used as catalyst in the oxidative zinc insertion into the Se-Se bond (Table 2, entries 3 and 4) and that the best results were obtained using TFA. When the reaction was performed using the TMEDA-stabilized zinc selenate **3**, we observed only a decrease of the reactivity, obtaining a good conversion of **4a** into **5a** (71%) in 30 min (Table 2, entry 11). Interestingly, the sulfur-containing reagent **2** afforded only 50% of conversion (Table 2, entry 12) and this is consistent with DFT calculations, that evidenced a higher reactivity of the sulfur reagents. This aspect, probably, represents a problem for the stability of the zinc thiolate during the solvent evaporation or in the presence of water, leading to a faster decomposition of the reactant.

From the results reported in Table 2, it appears clear that zinc-bis-chalcogenates are considerably more atom-efficient respect other similar reagents (compare entries 10,11 vs. 7,8; and 12 vs. 9) and this is an important parameter to be evaluated in terms of “greenness” of a synthetic process.

The best conditions optimized for **1** and **3** were applied to a series of acyl chlorides **4a**–**h**, affording the corresponding selenol esters **5a**–**h**; the conversion rate as well as the isolate yields are reported in Table 3. All the final products were fully characterized by GC-MS, ^1^H- and ^13^C-NMR after purification by flash chromatography (For ^1^H- and ^13^C-NMR of the purified compounds see Supplementary Materials).

Results collected in Table 3 indicate that the reaction works well with various acyl chlorides (**4a**–**h**), including aromatic and aliphatic ones, with the only exception of cinnamic derivative **4g**, that afforded only in traces of the corresponding selenol ester **5g** (Table 3, entry 7). In contrast to the previously described biphasic system [56], this approach tolerates functional groups sensitive to reduction, allowing the preparation of selenol ester **4d** (R=3,5(NO_2_)_2_C_6_H_3_) in excellent yields (Table 3, entry 4). Noteworthily, it can be observed that acyl chlorides bearing electron-withdrawing groups gave better yields, probably because of the more pronounced electrophilic character of the carboxylic carbon.

In order to clarify the nature of the species that are involved in the reaction mechanism leading to **5a**, the possible 1:1 initial intermediate model species formed by the interaction between (PhSe)_2_Zn **1** and PhCOCl **4a** was optimized. The only optimized geometry of the adducts shows PhCOCl interacting with the zinc atom of (PhSe)_2_Zn through its carbonyl group, thus distorting the Ch–Zn–Ch group towards a roughly trigonal geometry. A natural population analysis shows that the interaction results in a charge-transfer (CT) from PhCOCl to (PhSe)_2_Zn, whose LUMO is partly localized on the positively charged metal ion (Q_Zn_ = +0.857). The CT-interaction results in an increase in the positive charge on the acyl carbon and the polarization of the C=O bond (|Δ*Q*_CO_| = 1.083, 1.177 for PhCOCl; and PhSeZnSePh·PhCOCl, respectively). Therefore, the activated carbonyl group in PhCOCl might be more suitable to undergo a nucleophilic attack by the chalcogen donor of a second molecule of (PhCh)_2_Zn.

The TMEDA-stabilized reagent **3** was investigated using the same set of acyl chlorides **4a**–**h** and, generally, it confirmed a slightly/moderate reduced reactivity with a superimposable trend respect to the nature of the substrate. The only exception was observed for acyl chloride **4g**, that using TMEDA afforded selenol ester **5g** in 80% yield (Table 3, entry 7).

**Figure 4:**
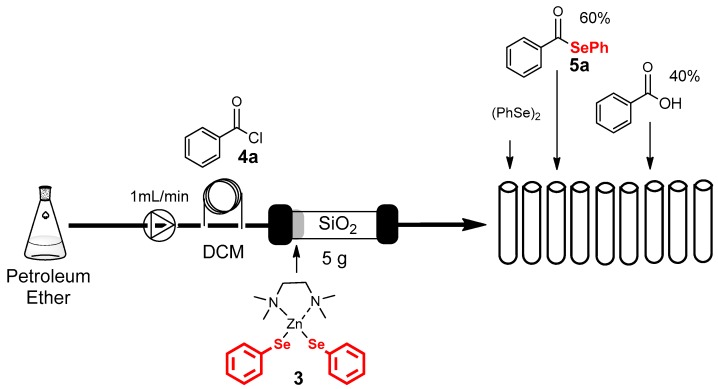
One-pot synthesis and chromatographic purification of the selenol ester **5a**.

Considering that the complex **3** is reported in literature as particularly stable and it is sufficiently soluble in several organic solvents, we envisioned the possibility to perform a ”one-pot” synthesis and chromatographic purification of the selenol ester **5a** following the setup reported in Figure 4. A pump fluxes petroleum ether through a Flash Pack Jones Chromatography apparatus silica gel column packed with 5 g of silica flash (40–63 µm) and, at the head of the column, was poured the reagent **3** (0.25 mmol), mixed with 500 mg of the same silica. The column was conditioned with the solvent and the substrate (0.5 mmol), dissolved in DCM, was injected. After the column, the eluent was fractioned and the fractions were isolated and characterized by NMR. The selenol ester **5a** was obtained as pure compound (separated from both, diphenyl diselenide and benzoic acid) in a yield comparable to that observed in the bench condition (60%). This protocol allowed to bypass the workup of the reaction saving a considerable amount of organic solvents (15 mL of EtOAc), brine solution (20 mL) and Na_2_SO_4,_ reducing the production of wastes.

## 3. Materials and Methods

Reactions were conducted in a round bottom flask and were stirred with Teflon-coated magnetic stirring bars at 800 rpm. Solvents and reagents were used as received unless otherwise noted. Analytical thin-layer chromatography (TLC) was performed on silica gel 60 F254 precoated aluminum foil sheets and visualized by UV irradiation or by KMnO_4_ staining. Kieselgel 60 (70–230 mesh) silica gel was used for column chromatography. NMR experiments were conducted at 25 °C with a DPX 200 spectrometer (Bruker, Faellanden, Switzerland).) operating at 200 MHz for ^1^H, 50.31 MHz for ^13^C experiments or with a Bruker DRX spectrometer (Bruker, Faellanden, Switzerland) operating at 400 MHz for ^1^H, 100.62 MHz for ^13^C and 76.31 MHz for ^77^Se. Chemical shifts (δ) are reported in parts per million (ppm), relative to TMS (δ = 0.0 ppm) and the residual solvent peak of CDCl_3_ (δ = 7.26 and 77.00 ppm in ^1^H- and ^13^C-NMR, respectively) and PhSeSePh δ = 464 ppm in ^77^Se. Data are reported as chemical shift (multiplicity, coupling constants where applicable, number of hydrogen atoms, and assignment where possible). Abbreviations are: s (singlet), d (doublet), t (triplet), q (quartet), quin (quintet), dd (doublet of doublet), dt (doublet of triplet), tt (triplet of triplet), m (multiplet), br. s (broad signal). Coupling constants (*J*) are quoted in Hertz (Hz) to the nearest 0.1 Hz. GC-MS analyses were carried out with an HP-6890 gas chromatography (dimethyl silicone column, 12.5 m) equipped with an HP-5973 mass-selective detector (Hewlett-Packard, Waldbronn, Germany). Acyl chlorides used for obtaining the selenol esters **5a**, **5e**, **5f** and **5h** are commercially available; acyl chlorides used for obtaining the selenol esters **5b**, **5c**, **5d** and **5g** were synthesized according to the literature [65]. Density Functional Theory (DFT) calculations were performed with the commercial suite of software Gaussian09 [66]. All calculations were carried out with the mPW1PW hybrid functional [67] and the full-electron Ahlrichs double-ζ basis sets with polarization functions (pVDZ) for all atomic species [67]. NBO populations [68,69,70] and Wiberg bond indices [71] were calculated at the optimized geometries, which were verified by harmonic frequency calculations. The results of the calculations were examined with GaussView 5 [72] and Molden 5.3 [73] programs.

### General Procedure for the Synthesis of Selenol Esters ***5a***–***h***

To a water suspension of the zinc complexes **1** or **3** (0.5 mmol, 6 mL of H_2_O), acyl chloride **4** (1 mmol) was added at room temperature and under stirring. After 30 min, the reaction mixture was extracted with ethyl acetate (3 × 10 mL), washed with brine (2 × 20 mL), dried with Na_2_SO_4_ and concentrated under vacuum to obtain a residue that was purified by flash chromatography.

*Se-Phenyl benzoselenoate* (**5a**) [40] was purified eluting with 20% DCM in petroleum ether. Yellow solid. m.p.: 39°–40 °C (Lit.: [38] 37°–38 °C). ^1^H-NMR (CDCl_3_, ppm) δ: 7.96–7.92 (m, 2H, H-Ar), 7.63–7.42 (m, 8H, H-Ar) ppm. ^13^C-NMR (CDCl_3_, ppm) δ: 193.5, 138.5, 136.4, 133.9, 129.4, 129.1, 128.9, 127.4, 125.8 ppm. CG-MS: *m*/*z* (%) = 262 (1) [M^+^], 157 (5), 105 (100), 77 (50), 51 (14).

*Se-Phenyl 2-bromobenzoselenoate* (**5b**) [74] was purified eluting with 5% EtOAc in petroleum ether. Yellow oil. ^1^H-NMR (CDCl_3_, ppm) δ: 7.72–7.6 (m, 4H, H-Ar), 7.45–7.34 (m, 5H, H-Ar) ppm. ^13^C-NMR (CDCl_3_, ppm) δ: 194.4, 140.6, 135.8, 134.3, 132.6, 129.5, 129.2, 128.8, 127.3, 126.6, 118.0 ppm.^77^Se-NMR (CDCl_3_, ppm) δ: 662.1 ppm. CG-MS *m*/*z* (%) = 340 (1) [M^+^], 232 (3), 183 (100), 157 (54), 76 (16), 50 (9).

*Se-Phenyl 4-butylbenzoselenoate* (**5c**) [50] was purified eluting with 20% DCM in petroleum ether. Yellow oil. ^1^H-NMR (CDCl_3_, ppm) δ: 7.85 (d, *J* = 8.1 Hz, 2H, H-Ar), 7.61–7.57 (m, 2H, H-Ar), 7.44–7.41 (m, 3H, H-Ar), 7.31–7.26 (m, 2H, H-Ar), 2.67 (t, *J* = 7,8 Hz, 2H, CH_2_), 1.61 (quin, *J* = 8.15 Hz, 2H, CH_2_), 1.36 (sex, *J* = 7.6 Hz, 2H, CH_2_), 0.95 (t, *J* = 7.2 Hz, 3H, CH_3_) ppm. ^13^C-NMR (CDCl_3_, ppm) δ: 192.7, 149.8, 136.4, 136.2, 129.3, 129.0, 127.5, 126.0, 35.8, 33.1, 22.3, 13.9 ppm. ^77^Se-NMR (CDCl_3_, ppm) δ: 661.0 ppm. CG-MS *m*/*z* (%) = 318 [M^+^], 161 (100), 91 (30).

*Se-Phenyl-3,5-dinitrobenzoselenoate* (**5d**) [50] was purified eluting with 5% EtOAc in petroleum ether. Yellow solid. m.p.: 148–150 °C (Lit.: [48] 148°–150°). ^1^H-NMR (CDCl_3_, ppm) δ: 9.28 (t, *J* = 2.05 Hz, 1H, H-Ar), 9.04 (d, *J* = 2.06 Hz, 2H, H-Ar), 7.7–7.4 (m, 5H, H-Ar) ppm. ^13^C-NMR (CDCl_3_, ppm) δ: 190.4, 148.9, 141.5, 136.1, 130.07, 129.9, 126,7, 124.1, 122.6 ppm. ^77^Se-NMR (CDCl_3_, ppm) δ: 662.3 ppm.

*Se-Phenyl 2-phenylethaneselenoate* (**5e**) [75] was purified eluting with 20% DCM in petroleum ether. Yellow solid. m.p.: 41–43 °C (Lit.: [59] 41°–43 °C). ^1^H-NMR (CDCl_3_, ppm) δ: 7.45–7.35 (m, 2H, H-Ar), 7.34–7.26 (m, 8H, H-Ar), 3.88 (s, 2H, CH_2_) ppm. ^13^C-NMR (CDCl_3_, ppm) δ: 198.9, 135.8, 132.6, 130.1, 129.3, 128.9, 128.8, 127.8, 126.6, 53.6 ppm. CG-MS *m*/*z* (%) = 276 [M^+^], 157 (22), 119 (26), 91 (100), 65 (26).

*Se-Phenyl thiophene-2-carboselenoate* (**5f**) [22] was purified eluting with 5% EtOAc in petroleum ether. Yellow oil. ^1^H-NMR (CDCl_3_, ppm) δ: 7.88 (dd, *J* = 1.2, 3.9 Hz, 1H, H-Ar), 7.71 (dd, *J* = 1.2, 4.96, Hz, 1H, H-Ar), 7.63–7.58 (m, 2H, H-Ar), 7.44–7.41 (m, 3H, H-Ar), 7.17 (dd, *J* = 3.9, 4.96 Hz, 1H, H-Ar) ppm. ^13^C-NMR (CDCl_3_, ppm) δ: 183.6, 143.1, 136.3, 133.7, 132.0, 129.4, 129.2, 128.0, 125.5 ppm. CG-MS *m*/*z* (%) = 268 (1) [M^+^], 157 (16), 111 (100), 83 (20).

*(E)-Se-Phenyl 3-phenylprop-2-eneselenoate* (**5g**) [65] was purified eluting with 5% EtOAc in petroleum ether. Yellow solid. m.p.: 79–80 °C (Lit.: [57] 81°–82 °C).^1^H-NMR (CDCl_3_, ppm) δ: 7.58–7.54 (m, 5H, H-Ar), 7.43–7.40 (m, 6H, H-Ar), 6.78 (d, *J* = 15.0 Hz, 1H, CH) ppm. ^13^C-NMR (CDCl_3_, ppm) δ: 190.8, 141.1, 135.9, 133.9, 130.9, 129.4, 129.1, 129.0, 128.6, 128.1, 126.3 ppm. ^77^Se-NMR (CDCl_3_, ppm) δ: 663.6 ppm. CG-MS *m*/*z* (%) = 288 (1) [M^+^], 157 (14), 131 (100), 103 (55), 77 (36).

*Se-Phenyl dodecaneselenoate* (**5h**) [76] was purified eluting with 20% DCM in petroleum ether. Yellow oil. ^1^H-NMR (CDCl_3_, ppm) δ: 7.55–7.53 (m, 2H, H-Ar), 7.42–7.39 (m, 3H, H-Ar), 2.73 (t, *J* = 7.5 Hz, 2H, CH_2_C(O)), 1.73 (quin, *J* = 7.4 Hz; 2H, CH_2_), 1.4–1.3 (m, 16H, CH_2_), 0.94–0.90 (t, *J* = 6.5 Hz, 3H, CH_3_) ppm. ^13^C-NMR (CDCl_3_, ppm) δ: 200.3, 135.7, 129.2, 128.7, 126.5, 47.5, 31.8, 29.5, 29.3, 29.26, 29.17, 28.8, 25.3, 22.6, 14.0 ppm. CG-MS *m*/*z* (%) = 340 (2) [M^+^], 183 (100), 157 (34), 109 (20), 85 (27), 71 (30), 57 (43).

## 4. Conclusions

In conclusion, we report here that the side reactivity of zinc bis-selenates with the solvent (THF) during the reaction with acyl chlorides can be rationalized by DFT calculations demonstrating that the interactions between (PhSe)_2_Zn species and the solvent units are remarkably stronger than those calculated between PhSeZnCl and PhSeZnBr and THF. Even if water seems to be equally able to coordinate the Zn, it was not possible to perform the oxidative zinc insertion directly in “on water conditions”. Nevertheless, after the removal of THF, the solid product obtained by the reduction of PhSeSePh with Zn in the presence of a catalytic amount of TFA (formally [(PhSe)_2_Zn]) can be efficiently used for the synthesis of selenol esters starting from the corresponding acyl chlorides and using water as reaction medium. Furthermore, the complex [(PhSe)_2_Zn]-TMEDA, prepared according to literature, showed a similar reactivity in water having the additional advantage to be bench stable. To the best of our knowledge, this is the first example that report the use of [(PhSe)_2_Zn]-TMEDA as nucleophilic reagent and the possibility to use it in a one pot reaction-purification process is an intriguing aspect that is currently under deep investigation by some of us. It is important to underline that the reagents reported in the present work (**1**, **2**, and **3**) are more atom-efficient not only relative to the previously reported zinc-halo-selenates, but also among most of the alternative known methods for the synthesis of selenol esters. During the preparation of this manuscript a further synthesis of these compounds appeared starting from anhydrides, evidencing the current interest in this class of derivatives [24]. Nevertheless, in our opinion, both in terms of atom economy and general applicability, it can be claimed that the use of acyl chloride results largely preferable if compared to the anhydrides.

## Figures and Tables

**Table 1:**
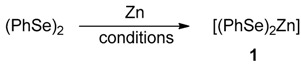
Synthesis of compound **1**.

Entry	Solvent	Additive	Time	T (°C)	Discoloration
1	H_2_O	none	2 h	70	No
2	THF	none	2 h	reflux	No
3	H_2_O	TFA 10 mol %	1 h	70	No
4	THF	TFA 10 mol %	20 min	reflux	Yes
5	H_2_O/THF	TFA 10 mol %	20 min	70	Yes

**Table 2:**
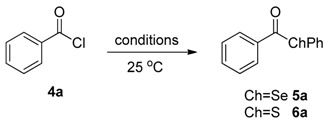
Zinc chalcogenates **1**–**3** in the preparation of chalcogenol esters.

Entry	Reagent	Medium	Time (h)	Yield (%) ^a^	ae (%) ^b^	Reference
1	PhSeZnCl	THF	24	25	66	[50]
2	PhSeZnBr	THF	24	30	60	[50]
3	[PhSeZnSePh] **1**	THF	3	32 ^c^	79.6	[56]
4	[PhSeZnSePh] **1**	THF	3	40 ^d^	79.6	–
5	PhSZnBr	THF	24	86	54.6	[57]
6	[PhSeZnSePh/PhSeH]	HCl_acq_/Et_2_O	4	38	–	[56]
7	PhSeZnCl	H_2_O	3	60	66	[50]
8	PhSeZnBr	H_2_O	3	70	60	[50]
9	PhSZnBr	H_2_O	3	65	54.6	[57]
10	[PhSeZnSePh] **1**	H_2_O	0.5	83	79.6	–
11	[PhSeZnSePh]TMEDA **3**	H_2_O	0.5	66	77	–
12	[PhSZnSPh] **2**	H_2_O	0.5	50	76	–

^a^ Conversion estimated by NMR; ^b^ Atom economy = m.w. of final product × 100/Σ (m.w. reactants); ^c^ **1** was prepared in the presence of 10 mol % of TfOH. Compound **5a** was formed together with 34% PhC(O)O(CH_2_)_4_SePh and 28% PhC(O)O(CH_2_)_4_ O(CH_2_)_4_SePh; ^d^ **1** was prepared in the presence of 10 mol % of TFA. Compound **5a** was formed together with 27% PhC(O)O(CH_2_)_4_SePh and 5% PhC(O)O(CH_2_)_4_ Cl.

**Table 3:**
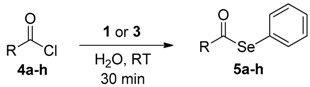
Synthesis of selenol esters **5**.

Entry	Substrate 4	Product 5	Conv, % of 4 in 5 (Yield, %) of 5 Using 1	Conv, % of 4 in 5 Using 3
1	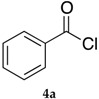	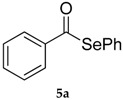	84 (80)	66
2	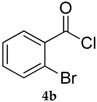	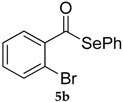	81 (53)	80
3	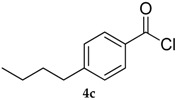	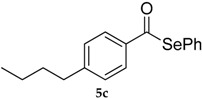	71 (57)	40
4	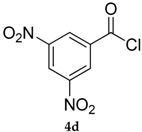	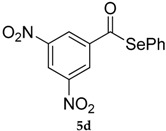	92 (90)	80
5	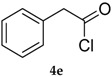	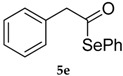	83 (64)	62
6	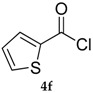	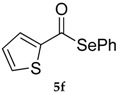	80 (65)	35
7	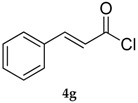	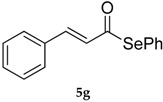	8	76
8	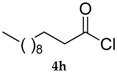	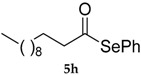	75 (69)	80

## Supplementary Materials

Copies of the ^1^H and ^13^C-NMR spectra are available online. Optimized geometries in orthogonal Cartesian format, Mulliken and Natural charges, and thermochemical data for all investigated compounds are available from M.A. upon request.
