# Supplementary material for: Atom Efficient Preparation of Zinc Selenates for the Synthesis of Selenol Esters under “On Water” Conditions
Source: Molecules. 2017 Jun 8;22(6):953. doi: 10.3390/molecules22060953 (PMC6152685; doi:10.3390/molecules22060953)
Supplement: Supplementary file 1 [file molecules-22-00953-s001.pdf]

Supplementary material

Atom Efficient preparation of Zinc Selenates for the Synthesis of Selenoesters under "*On Water*" Conditions

Luca Sancineto, Jaqueline Pinto Vargas, Bonifacio Monti, Massimiliano Arca, Vito Lippolis, Gelson Perin, Eder Joao Lenardao*, Claudio Santi*

Se-Phenyl benzoselenoate **(5a)** [1] purified eluting with 20 % DCM in petroleum ether. Yellow solid. M.p.: 39°-40°C (Lit.:[38] 37°-38°C). ^{1}H-NMR ($CDCl_3$, 200 MHz, ppm) δ: 7.96-7.92 (m, 2H, H-Ar), 7.63-7.42 (m, 8H, H-Ar) ppm. ^{13}C-NMR ($CDCl_3$, 50 MHz, ppm) δ: 193.5, 138.5, 136.4, 133.9, 129.4, 129.1, 128.9, 127.4, 125.8 ppm. CG-MS: *m/z* (%)= 262 (1) [M$^+$], 157 (5), 105 (100), 77 (50), 51 (14).

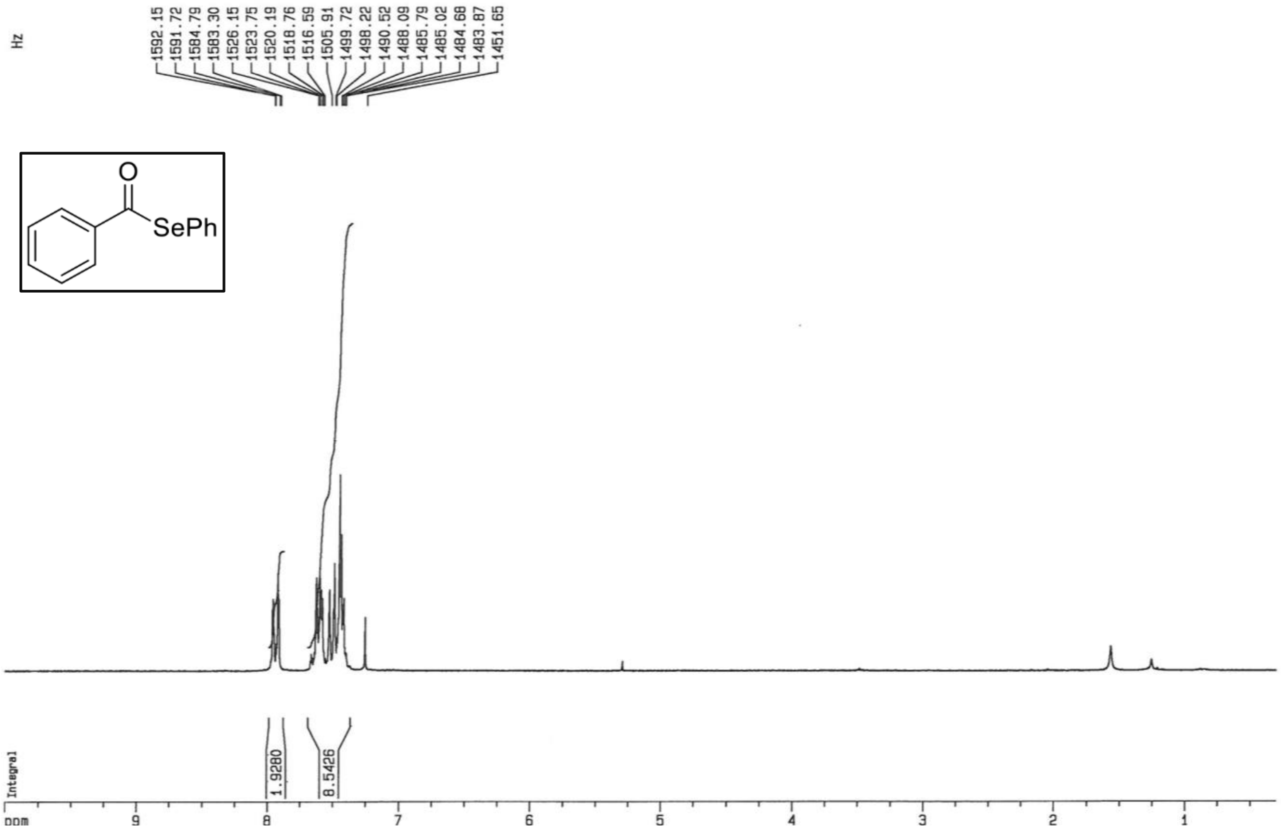

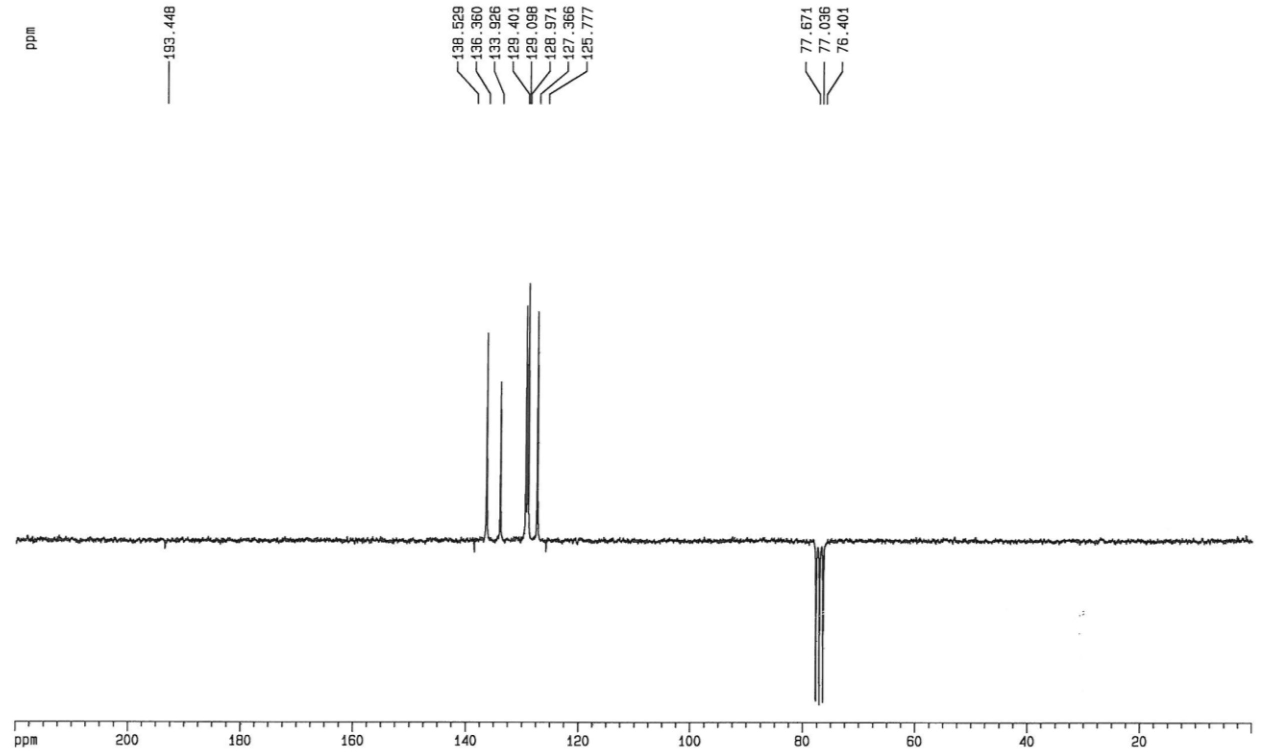

Se-Phenyl 2-bromobenzoselenoate **(5b)** [2] purified eluting with 5 % EtOAc in petroleum ether. Yellow oil. ^{1}H-NMR ($CDCl_3$, 200 MHz, ppm) δ: 7.72-7.6 (m, 4H, H-Ar), 7.45-7.34 (m, 5H, H-Ar) ppm. ^{13}C-NMR ($CDCl_3$, 50 MHz, ppm) δ: 194.4, 140.6, 135.8, 134.3, 132.6, 129.5, 129.2, 128.8, 127.3, 126.6, 118.0 ppm. CG-MS *m/z* (%)= 340 (1) [M$^+$], 232 (3), 183 (100), 157 (54), 76 (16), 50 (9).

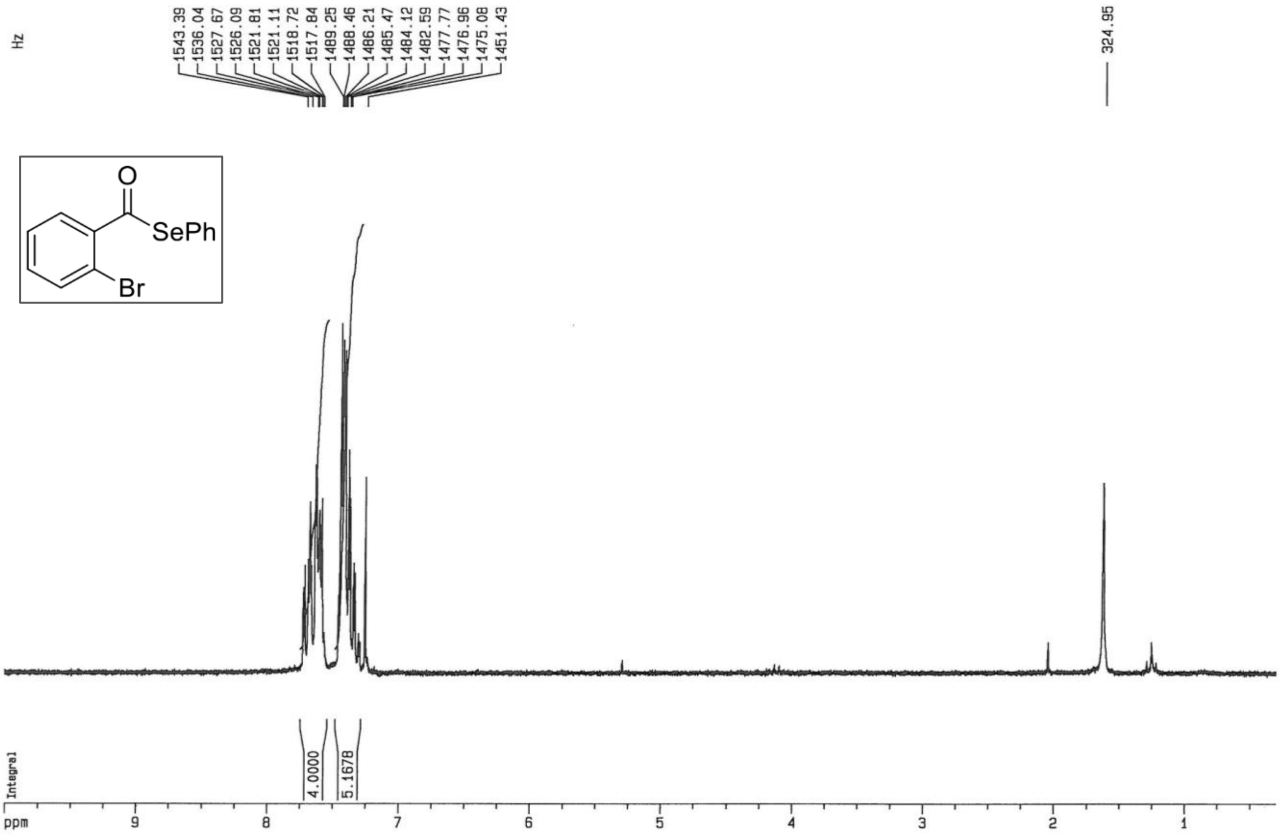

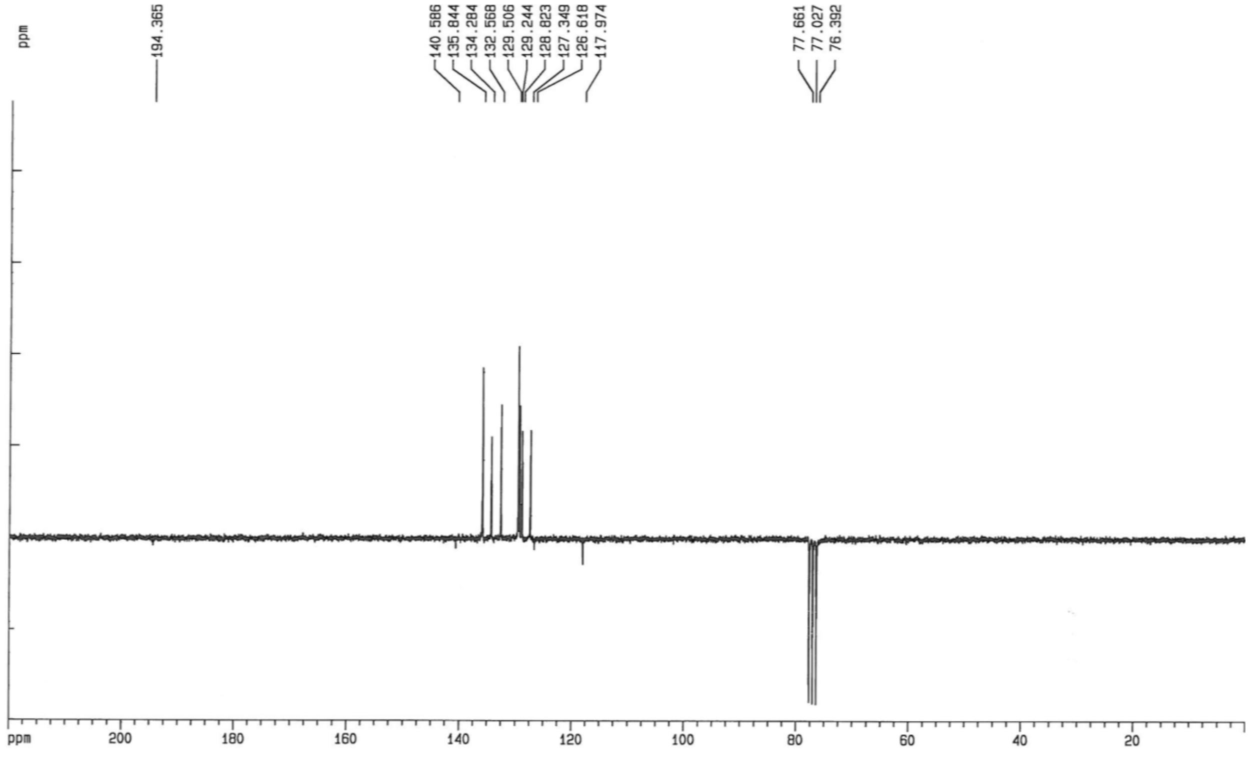

Se-Phenyl 4-butylbenzoselenoate **(5c)** [3] purified eluting with 20 % DCM in petroleum ether. Yellow oil. ^{1}H-NMR ($CDCl_3$, 200 MHz, ppm) δ: 7.85 (d, *J*= 8.1 Hz, 2H, H-Ar), 7.61-7.57 (m, 2H, H-Ar), 7.44-7.41 (m, 3H, H-Ar), 7.31-7.26 (m, 2H, H-Ar), 2.67 (t, *J*= 7,8 Hz, 2H, CH_2), 1.61 (quin, *J*= 8.15 Hz, 2H, CH_2), 1.36 (sex, *J*= 7.6 Hz, 2H, CH_2), 0.95 (t, *J*= 7.2 Hz, 3H, CH_3) ppm. ^{13}C-NMR ($CDCl_3$, 50 MHz, ppm) δ: 192.7, 149.8, 136.4, 136.2, 129.3, 129.0, 127.5, 126.0, 35.8, 33.1, 22.3, 13.9 ppm. CG-MS *m/z* (%)= 318 [M^+], 161 (100), 91 (30).

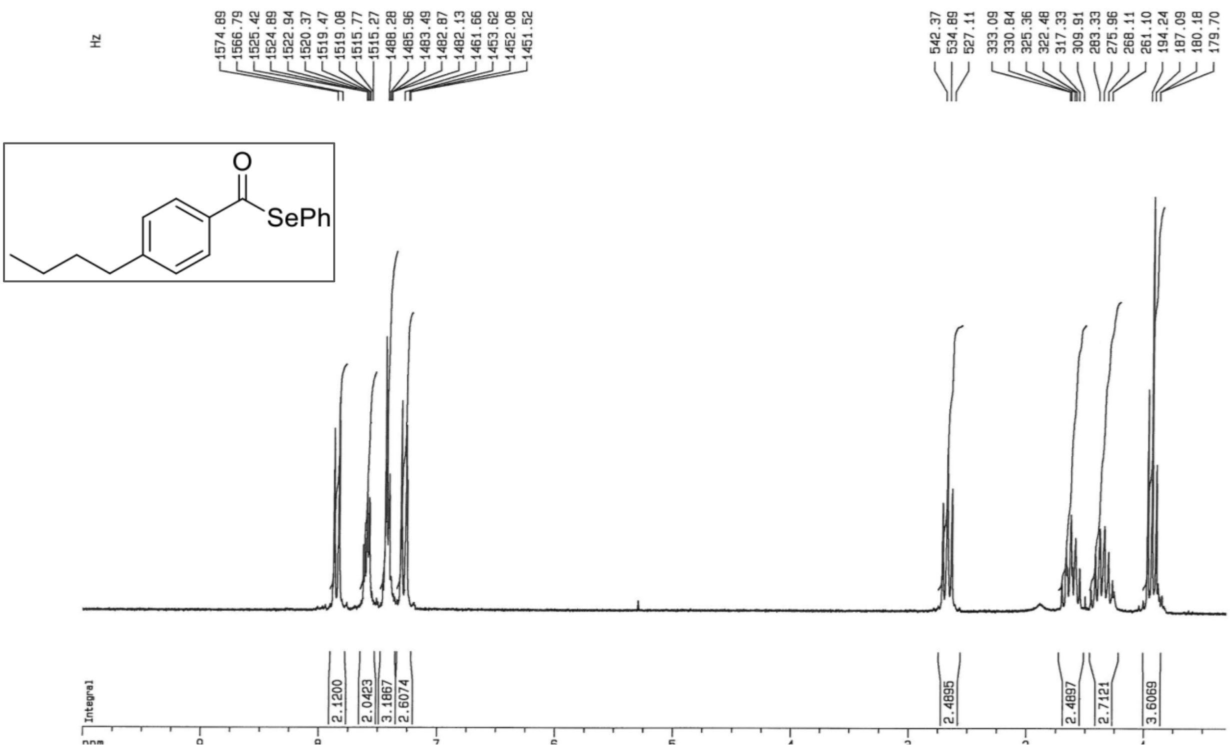

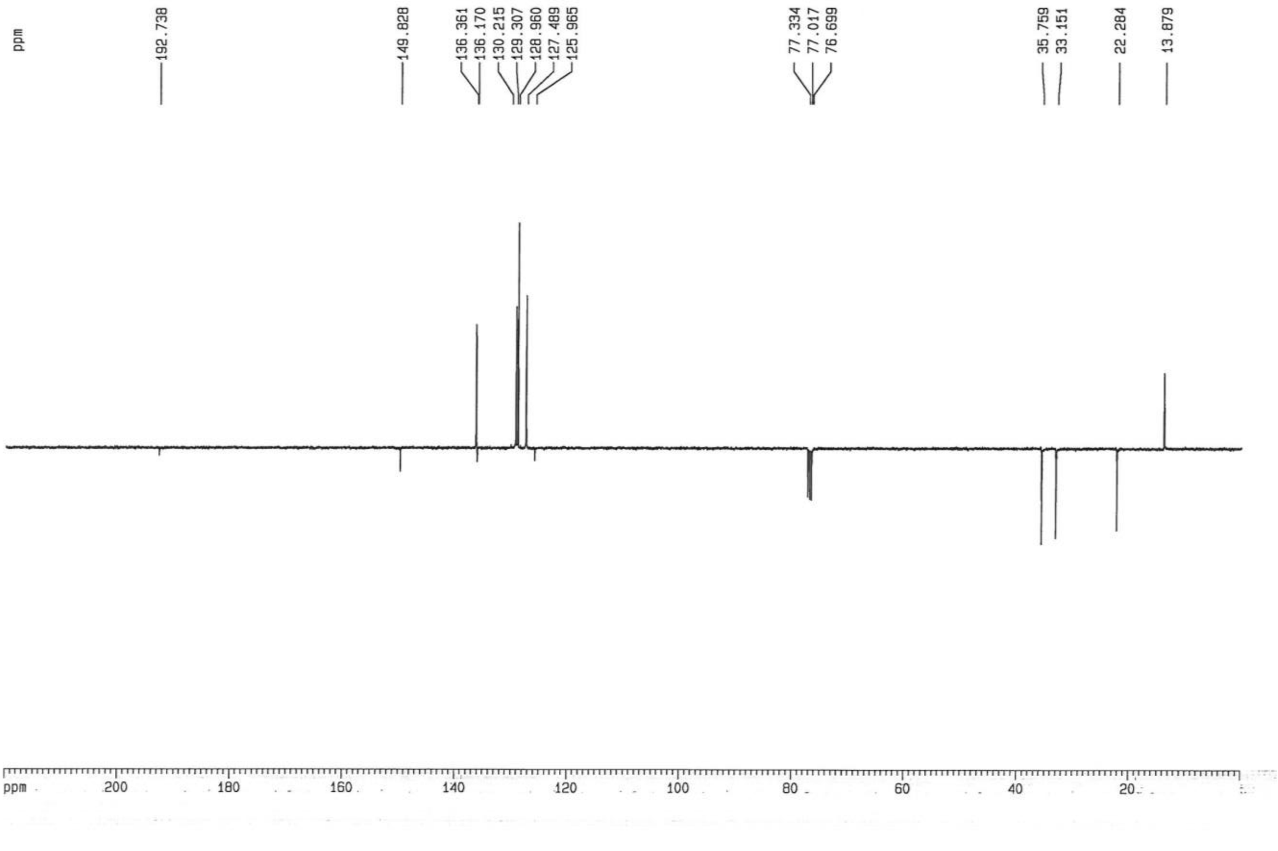

Se-Phenyl-3,5-dinitrobenzoselenoate **(5d)** [3] purified eluting with 5 % EtOAc. Yellow solid. M.p.: 148-150°C ((Lit.:[3] 148°-150°). ^{1}H-NMR ($CDCl_3$, 200 MHz, ppm) δ: 9.28 (t, *J*= 2.05 Hz, 1H, H-Ar), 9.04 (d, *J*= 2.06 Hz, 2H, H-Ar), 7.7-7.4 (m, 5H, H-Ar) ppm. ^{13}C-NMR ($CDCl_3$, 50 MHz, ppm) δ: 190.4, 148.9, 141.5, 136.1, 130.07, 129.9, 126,7, 124.1, 122.6 ppm.

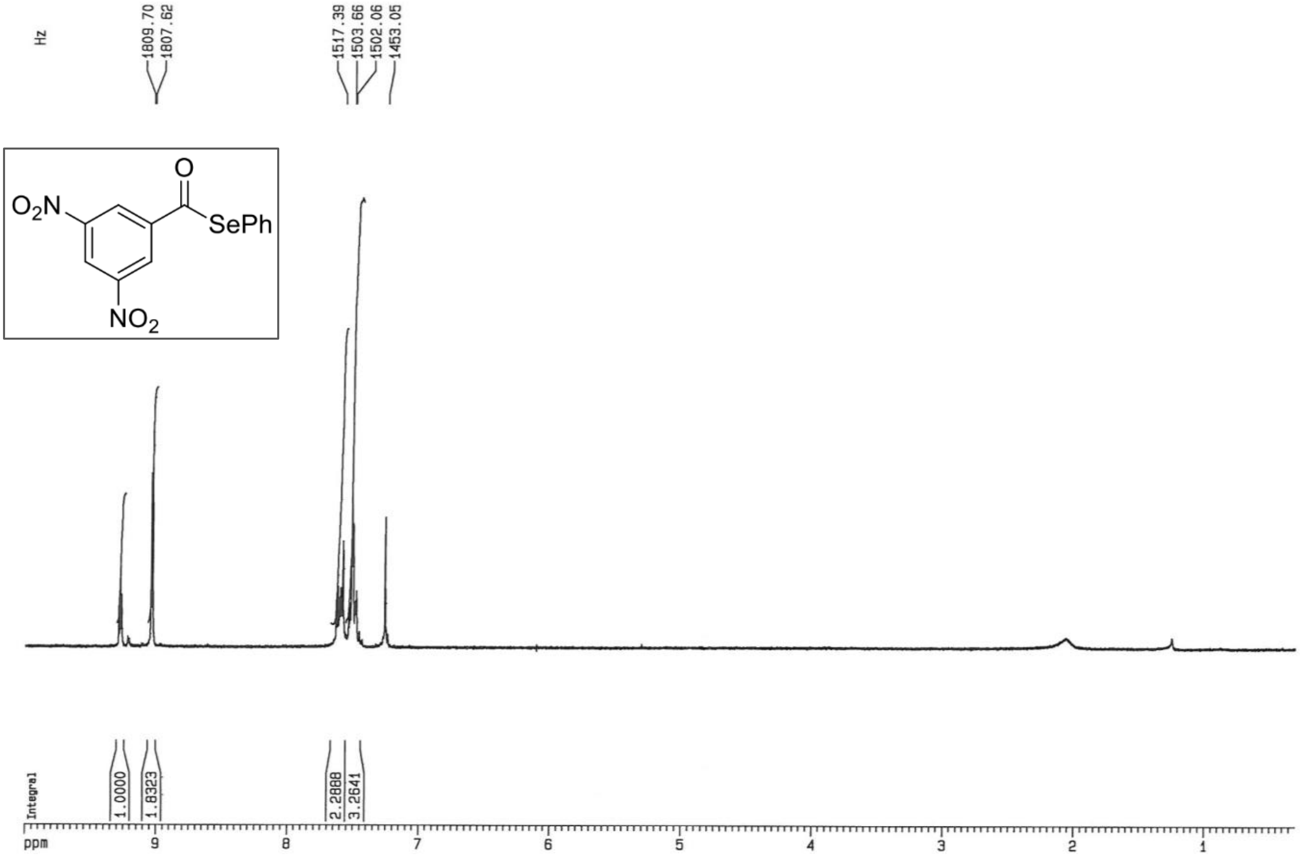

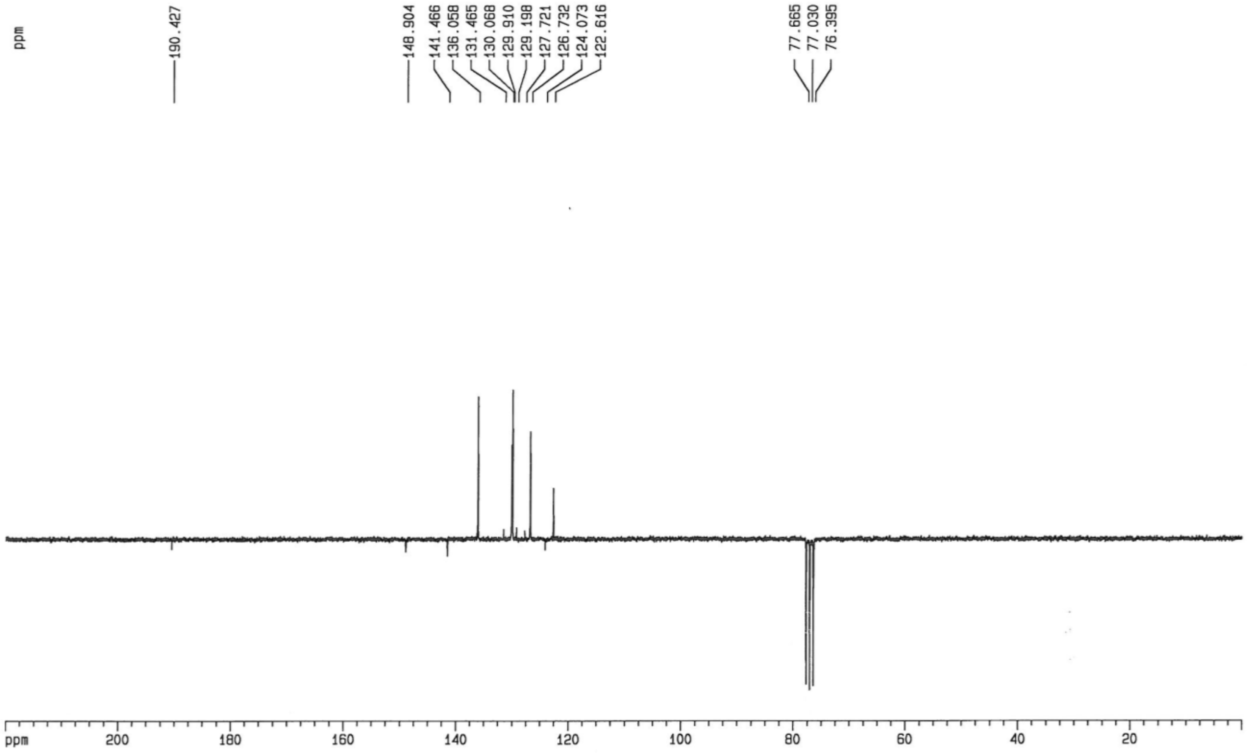

Se-Phenyl 2-phenylethaneselenoate **(5e)** [4] purified eluting with 20 % DCM in petroleum ether. Yellow solid. M.p.: 41-43°C ((Lit.[4]: 41°-43°C). ^{1}H-NMR ($CDCl_3$, 200 MHz, ppm) δ: 7.45-7.35 (m, 2H, H-Ar), 7.34-7.26 (m, 8H, H-Ar), 3.88 (s, 2H, CH_2) ppm. ^{13}C-NMR ($CDCl_3$, 50 MHz, ppm) δ: 198.9, 135.8, 132.6, 130.1, 129.3, 128.9, 128.8, 127.8, 126.6, 53.6 ppm. CG-MS *m/z* (%)= 276 [M$^+$], 157 (22), 119 (26), 91 (100), 65 (26).

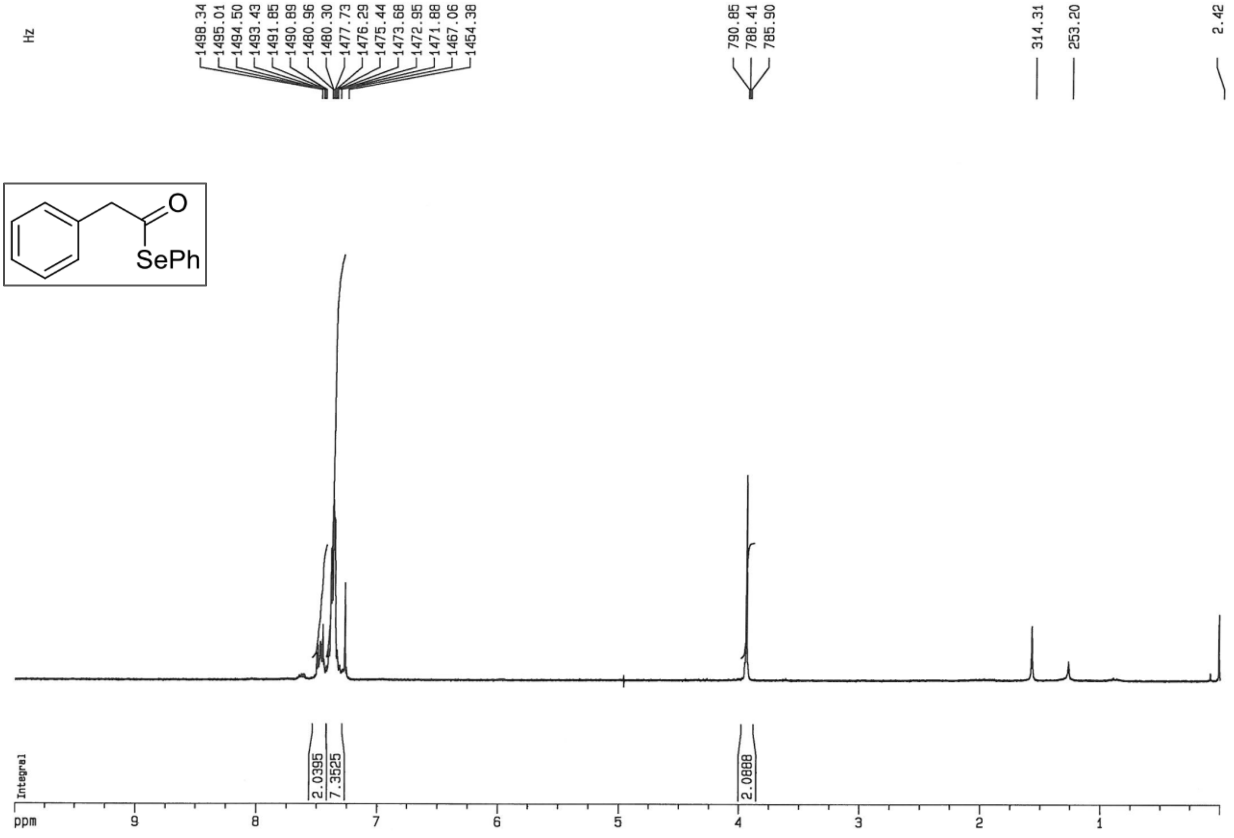

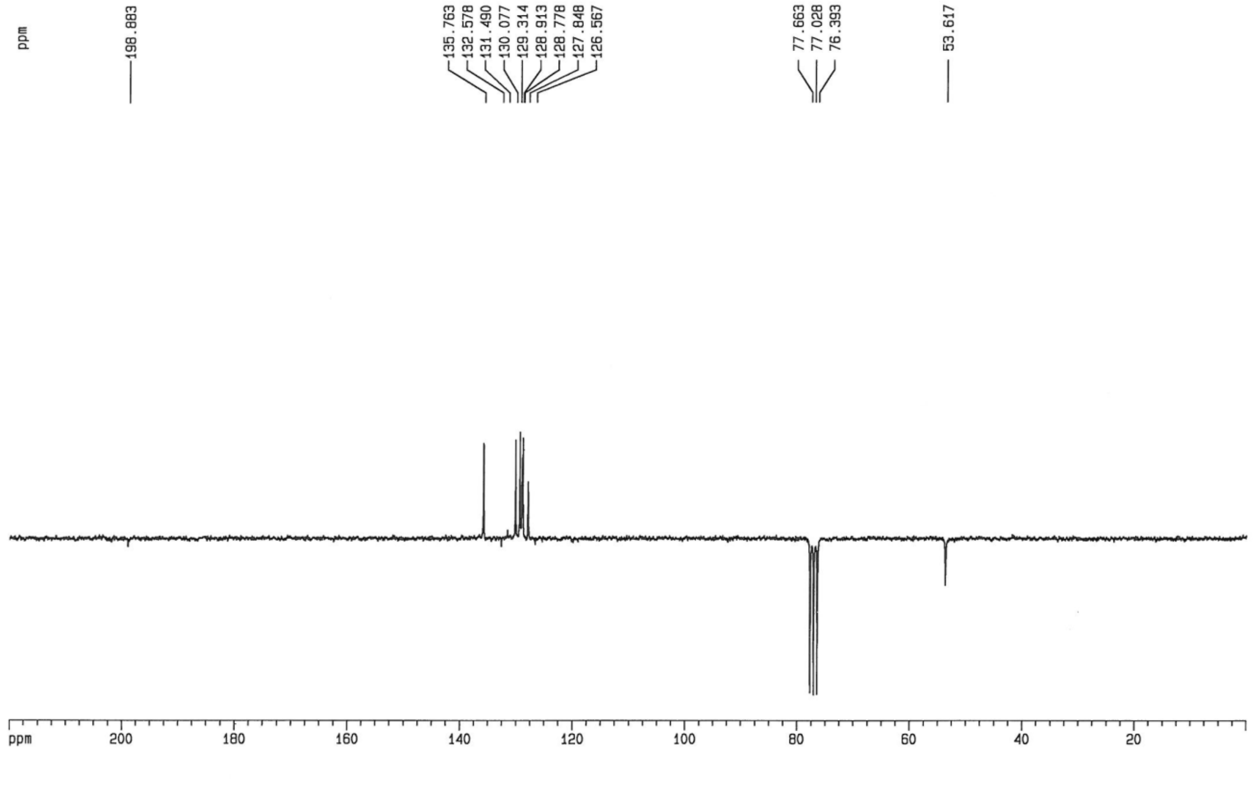

Se-Phenyl thiophene-2-carboselenoate **(5f)** [5] purified eluting with 5 % EtOAc. Yellow oil. ^{1}H-NMR ($CDCl_3$, 200 MHz, ppm) δ: 7.88 (dd, *J*= 1.2, 3.9 Hz, 1H, H-Ar), 7.71 (dd, *J*= 1.2, 4.96, Hz, 1H, H-Ar), 7.63-7.58 (m, 2H, H-Ar), 7.44-7.41 (m, 3H, H-Ar), 7.17 (dd, *J*= 3.9, 4.96 Hz, 1H, H-Ar) ppm. ^{13}C-NMR ($CDCl_3$, 50 MHz, ppm) δ: 183.6, 143.1, 136.3, 133.7, 132.0, 129.4, 129.2, 128.0, 125.5 ppm. CG-MS *m/z* (%)= 268 (1) [M+], 157 (16), 111 (100), 83 (20).

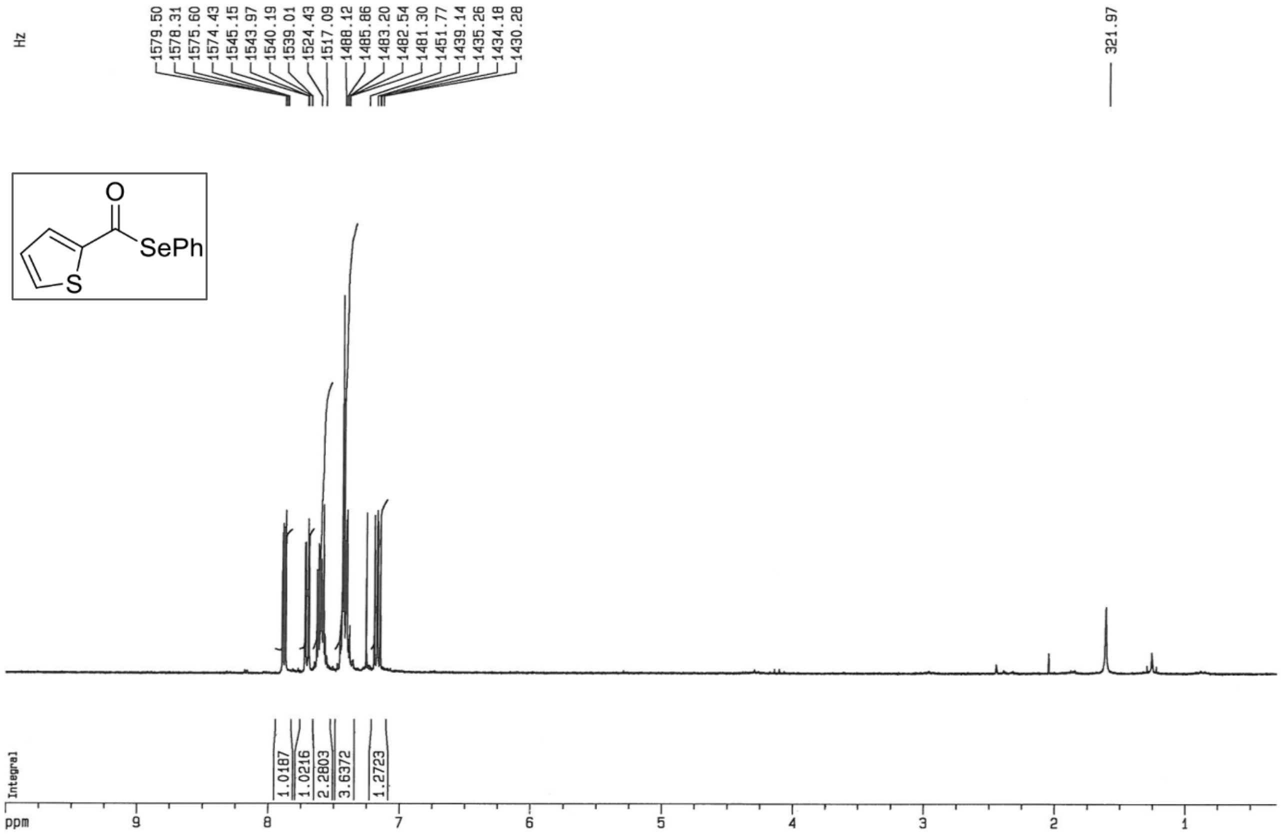

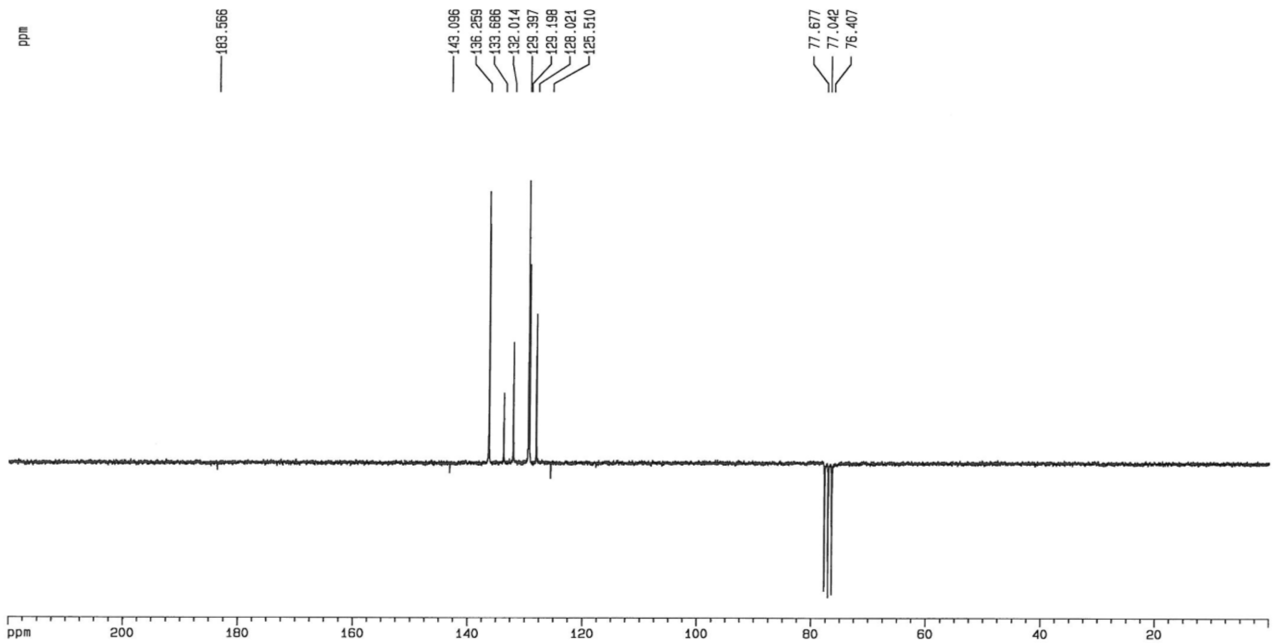

(E)-Se-Phenyl 3-phenylprop-2-eneselenoate **(5g)** [6] purified eluting with 5 % EtOAc. Yellow solid. P.f.: 79-80°C ((Lit.:[6] 81°-82°C).^{1}H-NMR ($CDCl_3$, 200 MHz, ppm) δ: 7.58-7.54 (m, 5H, H-Ar), 7.43-7.40 (m, 6H, H-Ar), 6.78 (d, *J*= 15.0 Hz, 1H, CH) ppm. ^{13}C-NMR ($CDCl_3$, 50 MHz, ppm) δ: 190.8, 141.1, 135.9, 133.9, 130.9, 129.4, 129.1, 129.0, 128.6, 128.1, 126.3 ppm. CG-MS *m/z* (%)= 288 (1) [M+], 157 (14), 131 (100), 103 (55), 77 (36).

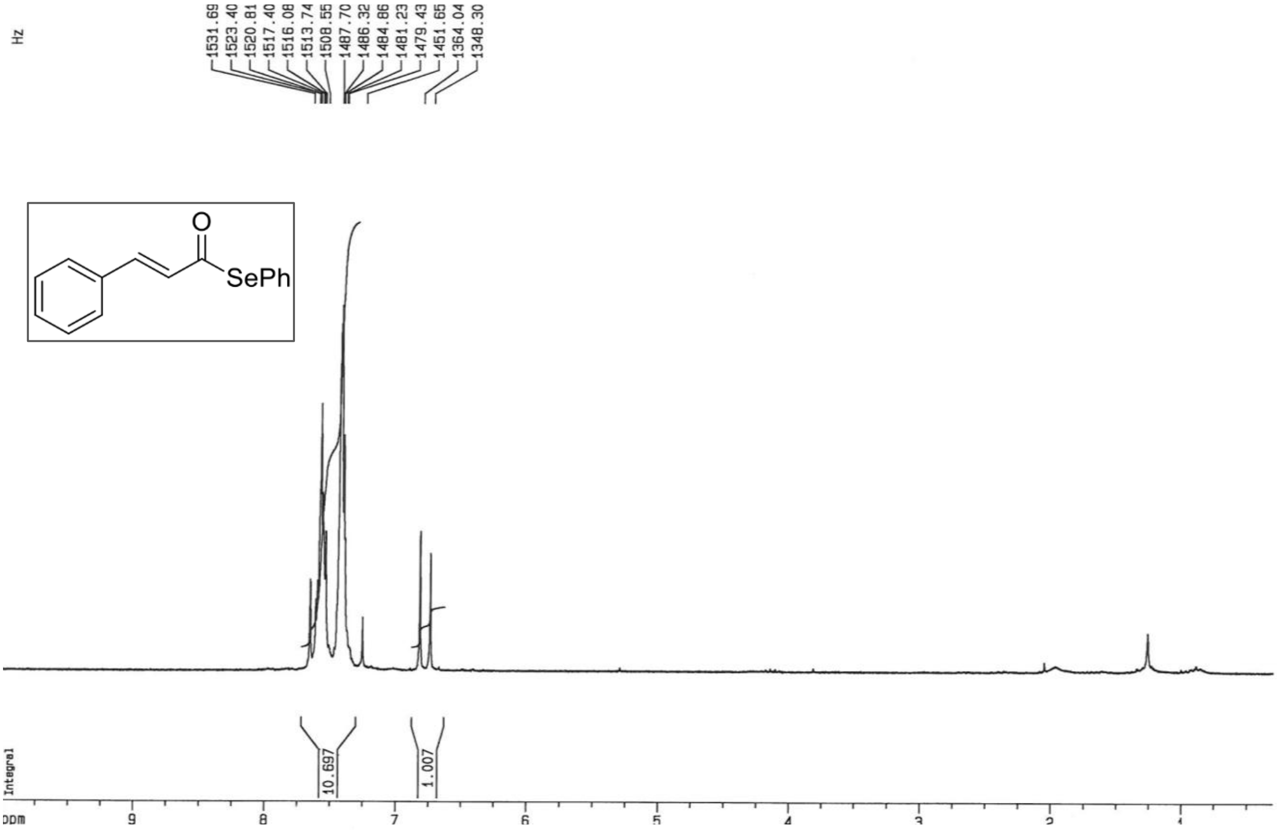

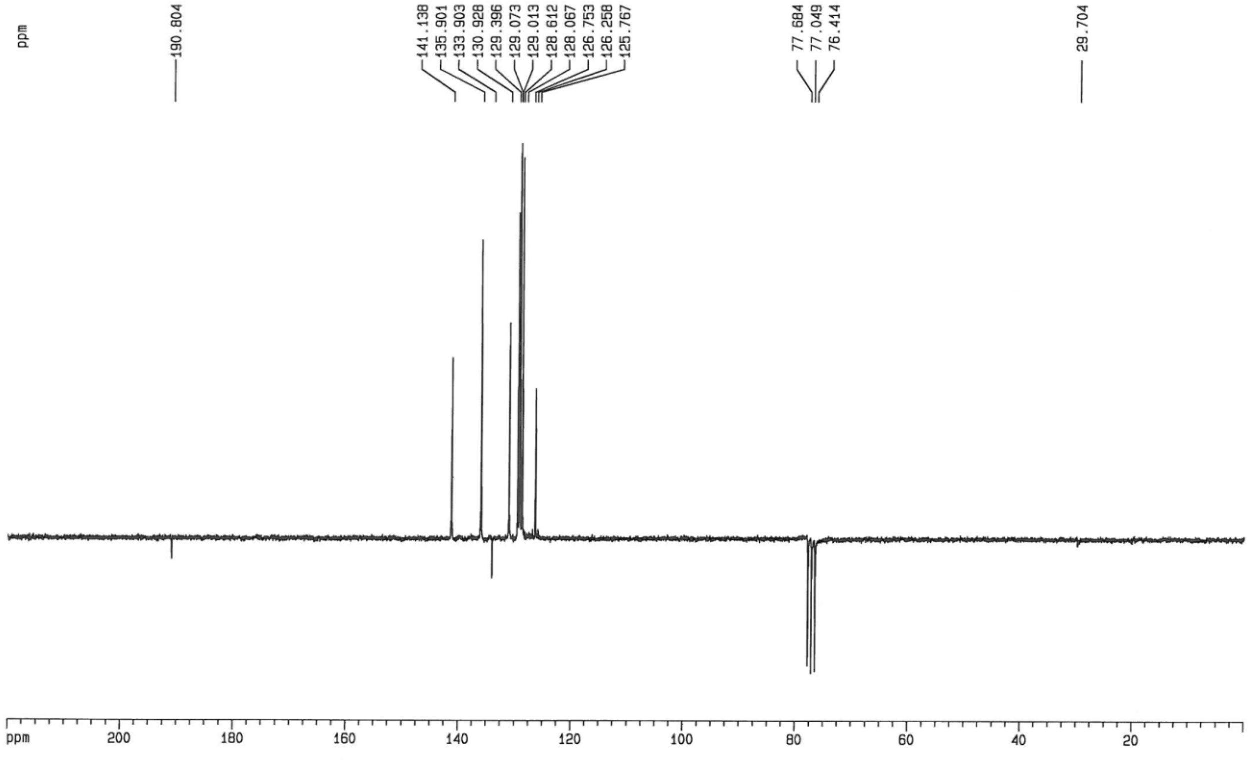

Se-Phenyl dodecaneselenoate (**5h**) [7] purified eluting with 20 % DCM in petroleum ether. Yellow oil. ^{1}H-NMR ($CDCl_3$, 400 MHz, ppm) δ: 7.55-7.53 (m, 2H, H-Ar), 7.42-7.39 (m, 3H, H-Ar), 2.73 (t, *J*= 7.5 Hz, 2H, $CH_2C(O)$), 1.73 (quin, *J*= 7.4 Hz; 2H, CH_2), 1.4-1.3 (m, 16H, CH_2), 0.94-0.90 (t, *J*= 6.5 Hz, 3H, CH_3) ppm. ^{13}C-NMR ($CDCl_3$, 100 MHz, ppm) δ: 200.3, 135.7, 129.2, 128.7, 126.5, 47.5, 31.8, 29.5, 29.3, 29.26, 29.17, 28.8, 25.3, 22.6, 14.0 ppm. CG-MS *m/z* (%)= 340 (2) [M+], 183 (100), 157 (34), 109 (20), 85 (27), 71 (30), 57 (43).

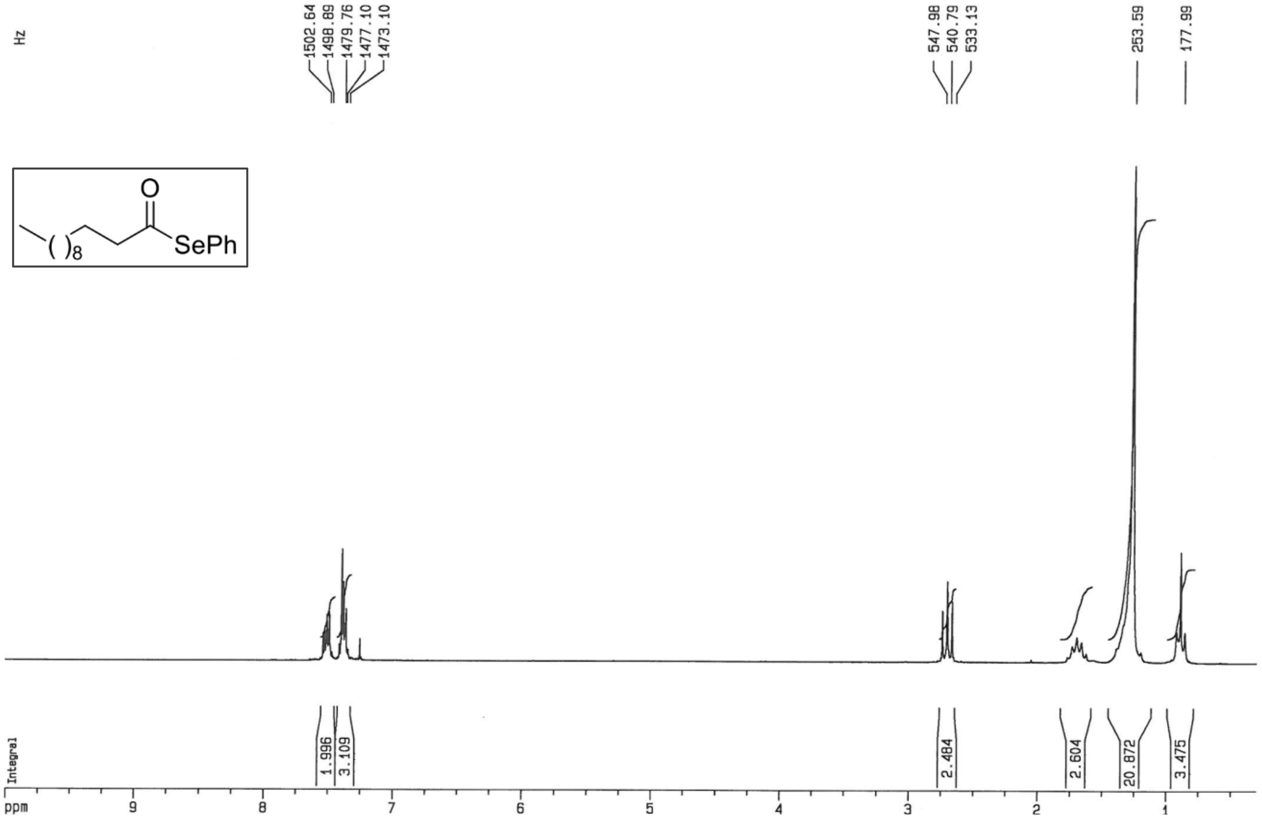

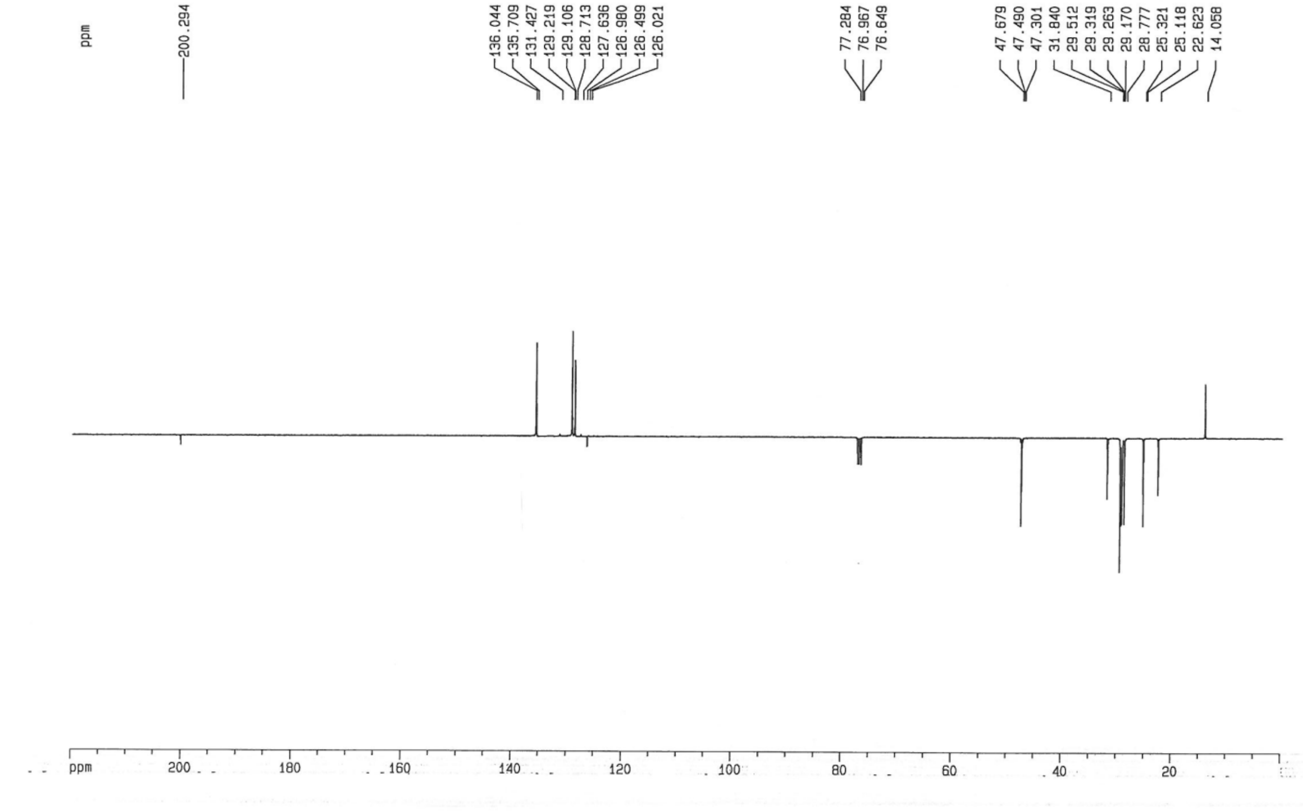

References

1. Marin, G.; Braga, A. L.; Rosa, A. S.; Galetto, F. Z.; Burrow, R. A.; Gallardo, H.; Paixão, M. W. Efficient synthesis of selenol esters from acid chlorides mediated by indium metal. *Tetrahedron* **2009**, *65*, 4614–4618.
2. Wang, L.; Zhang, Y. Water Accelerated Sm/TMSCl Reductive Cleavage of the Se-Se Bond: Synthesis of Selenoesters and Selenoformates. *Synth. Commun.* **1999**, *29*, 3107–3115.
3. Santi, C.; Battistelli, B.; Testaferri, L.; Tiecco, M. On water preparation of phenylselenoesters. *Green Chem.* **2012**, *14*, 1277.
4. Ren, K.; Wang, M.; Liu, P.; Wang, L. Iron-Catalyzed Synthesis of Selenoesters from Diselenides and Acyl Chlorides or Acid Anhydrides in the Presence of Magnesium Dust. *Synthesis (Stuttg).* **2010**, *2010*, 1078–1082.
5. Dan, W.; Deng, H.; Chen, J.; Liu, M.; Ding, J.; Wu, H. A new odorless one-pot synthesis of thioesters and selenoesters promoted by Rongalite®. *Tetrahedron* **2010**, *66*, 7384–7388.
6. Kudelko, A.; Wróblowska, M. An efficient synthesis of conjugated 5-aryl-1,3,4-oxadiazoles from 3-heteroarylacrylohydrazides and acid chlorides. *Tetrahedron Lett.* **2014**, *55*, 3252–3254.
7. Zhang, S.; Zhang, Y. Reductive Cleavage of Se–Si Bond in Arylselenotrimethylsilane: A Novel Method for the Synthesis of Selenoesters. *Synth. Commun.* **1998**, *28*, 3999–4002
